# Improved risk stratification by PET-based intratumor heterogeneity in children with high-risk neuroblastoma

**DOI:** 10.3389/fonc.2022.896593

**Published:** 2022-10-24

**Authors:** Chao Li, Shaoyan Wang, Can Li, Yafu Yin, Fang Feng, Hongliang Fu, Hui Wang, Suyun Chen

**Affiliations:** ^1^ Department of Nuclear Medicine, Xinhua Hospital Affiliated to Shanghai Jiao Tong University School of Medicine, Shanghai, China; ^2^ Department of Pathology, Xinhua Hospital Affiliated to Shanghai Jiao Tong University School of Medicine, Shanghai, China

**Keywords:** pediatric, neuroblastoma, ^18^F-FDG, PET/CT, intratumor heterogeneity, radiomics

## Abstract

**Purpose:**

The substratification of high-risk neuroblastoma is challenging, and new predictive imaging biomarkers are warranted for better patient selection. The aim of the study was to evaluate the prognostic role of PET-based intratumor heterogeneity and its potential ability to improve risk stratification in neuroblastoma.

**Methods:**

Pretreatment ^18^F-FDG PET/CT scans from 112 consecutive children with newly diagnosed neuroblastoma were retrospectively analyzed. The primary tumor was segmented in the PET images. SUVs, volumetric parameters including metabolic tumor volume (MTV) and total lesion glycolysis (TLG), and texture features were extracted. After the exclusion of imaging features with poor and moderate reproducibility, the relationships between the imaging indices and clinicopathological factors, as well as event-free survival (EFS), were assessed.

**Results:**

The median follow-up duration was 33 months. Multivariate analysis showed that PET-based intratumor heterogeneity outperformed clinicopathological features, including age, stage, and MYCN, and remained the most robust independent predictor for EFS [training set, hazard ratio (HR): 6.4, 95% CI: 3.1–13.2, *p* < 0.001; test set, HR: 5.0, 95% CI: 1.8–13.6, *p* = 0.002]. Within the clinical high-risk group, patients with a high metabolic heterogeneity showed significantly poorer outcomes (HR: 3.3, 95% CI: 1.6–6.8, *p* = 0.002 in the training set; HR: 4.4, 95% CI: 1.5–12.9, *p* = 0.008 in the test set) compared to those with relatively homogeneous tumors. Furthermore, intratumor heterogeneity outran the volumetric indices (MTVs and TLGs) and yielded the best performance of distinguishing high-risk patients with different outcomes with a 3-year EFS of 6% vs. 47% (*p* = 0.001) in the training set and 9% vs. 51% (*p* = 0.004) in the test set.

**Conclusion:**

PET-based intratumor heterogeneity was a strong independent prognostic factor in neuroblastoma. In the clinical high-risk group, intratumor heterogeneity further stratified patients with distinct outcomes.

## Introduction

Neuroblastoma is the most common extracranial solid tumor in children and is remarkable for its heterogeneity (1). Risk stratification using a combination of clinical and biological factors, such as age at diagnosis, stage, histology, and MYCN status, is of paramount importance to effectively inform therapeutic approaches. At the time of presentation, about 60% of children are classified as high risk (2). The incorporation of intensive multimodality therapy has increased the 5-year survival for high-risk neuroblastoma from less than 20% to ~50% (3). However, a notable subset of patients do not respond to induction therapy and have a dismal outcome, with a long-term survival of less than 15% (4). The improved outcome for the survivors has come at a cost of significant early or long-term toxicity. The early identification of these different subsets of patients may facilitate a more precisely tailored treatment, which remains an important unmet need.

Intratumor heterogeneity, resulting from subclonal genetic diversity within a tumor, manifests in spatial variation in stromal architecture and consumption of oxygen and glucose (5). It has been associated with poor prognosis and predisposes patients to inferior response to anticancer therapies (6). Medical images can depict the spatial heterogeneity in individual tumors andquantify the overall functional characteristics. Various approaches for the assessment of intratumor heterogeneity in PET images have been investigated, including simple visual analysis, histogram quantifying voxel distributions, and texture features quantifying spatial complexity (7, 8). A growing body of evidence suggests that PET-based intratumor heterogeneity might have predictive or prognostic value in various malignancies (9, 10).

^123^I-meta-iodobenzylguanidine (mIBG) scan has been the main imaging modality for neuroblastoma. For high-risk diseases, however, the limited prognostic value of pretreatment mIBG score was reported (11, 12). On the other hand, ^18^F-FDG PET/CT is increasingly used in neuroblastoma, particularly in tumors not taking up mIBG. SUVmax has been reported to correlate with MYCN amplification (13) and may serve as a prognostic biomarker in neuroblastoma (14, 15). Volumetric parameters derived from ^18^F-FDG PET, including metabolic tumor volume (MTV) and total lesion glycolysis (TLG), were previously reported as significant prognostic factors in neuroblastoma (16). To date, there is limited evidence regarding the role of intratumor metabolic heterogeneity in neuroblastoma.

Our key objectives were to investigate the prognostic role of PET-based intratumor heterogeneity and whether it could be used to further risk-stratify neuroblastoma.

## Materials and methods

### Patients

**Figure 1:**
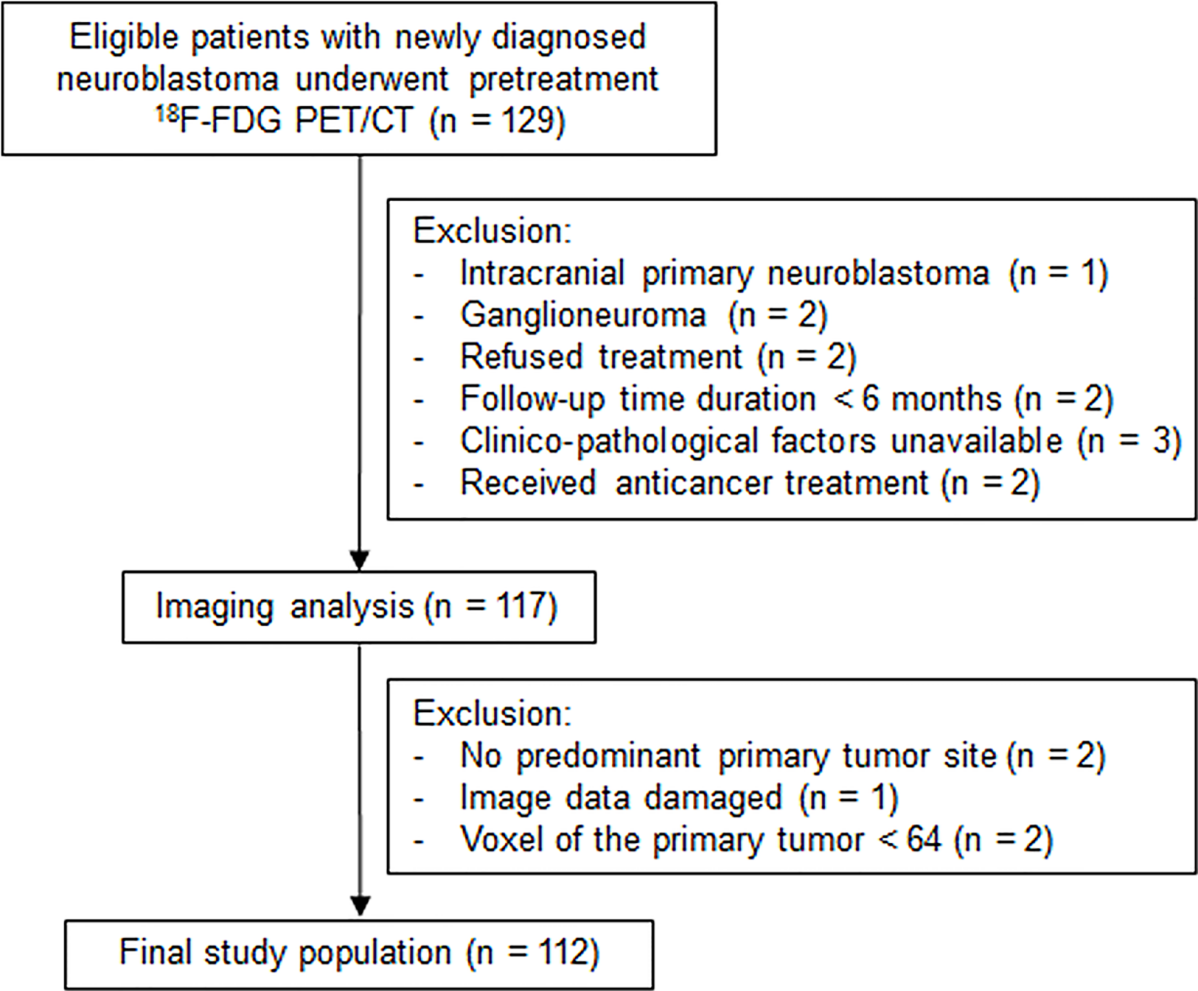
Flowchart shows study population selection, with exclusion criteria.

This study included 129 consecutive pediatric patients with histologically proven neuroblastoma between October 2011 and September 2020. The inclusion criteria were as follows: 1) newly diagnosed neuroblastoma with no previous anticancer treatment, 2) underwent baseline ^18^F-FDG PET/CT scan, 3) not accompanied by other malignancies, and 4) at least 6 months of follow-up. Patients were excluded if they had primary intracranial neuroblastoma, ganglioneuroma, no predominant primary tumor site, refused treatment, or had received chemotherapy before the PET scan (**Figure 1**). Clinicopathological prognostic indices, such as age, stage, risk stratification, MYCN, lactate dehydrogenase (LDH), and ferritin, were collected. This retrospective study was approved by the institutional review board, and the requirement for informed consent was waived.

### PET/CT imaging

^18^F-FDG was administered at a dose of 5.18 MBq/kg after at least 4–6 h of fasting. PET/CT scans from the skull to the proximal thigh were acquired about 60 min after injection using a Biograph mCT-64 scanner (Siemens). When metastasis was suspected to involve the extremities, imaging from the vertex to the toes including the arms was performed. Chloral hydrate sedation (50 mg/kg) was used 30 min before scanning for children unable to follow instructions. PET images were reconstructed using 3D ordered subset expectation maximization (3 iterations, 24 subsets). CT scans were acquired with 100-kV tube voltage, automated tube current modulation, 3-mm slice thickness, and a pitch of 1.5.

### Imaging segmentation and feature extraction

Segmentation and feature extraction were performed using the LIFEx software (Version 6.31, http://www.lifexsoft.org). To investigate the voxel relationships inside the entire tumor, volumes of interest (VOIs) covering the whole primary tumor were delineated manually in PET images by a nuclear medicine physician with more than 11 years of PET/CT experience without knowledge of clinical information. In some cases, the primary tumor fused with the metastatic lesions and was delineated with reference to recent contrast-enhanced CT or MRI images. As PET has relatively large voxels compared with CT and MRI, each VOI must contain at least 64 contiguous voxels according to the LIFEx user guide. Two patients were excluded due to small voxels. Imaging indices were computed after a resampling step using 64 bins (size bin of 0.3) without spatial resampling. MTV and TLG with a threshold of 41% of SUVmax (MTV41%, TLG41%), which has been reported to correspond best with the actual dimensions of the tumor for tumor boundary delineation (17), were extracted from the same VOIs.

### Clinical endpoints and risk stratification

Event-free survival (EFS) was calculated as the time from the start date of cancer treatment to the date of relapse, progression, or death from any cause. All the patients received risk-adapted treatment according to the Chinese Children Cancer Group-NB-2009/2014. The risk categorization schema was consistent with the Children’s Oncology Group protocol (2). Briefly, patients were classified into low-, intermediate-, and high-risk categories based on age, stage, and other histopathological factors. High-risk disease was defined as ≥18 months of age and either disseminated disease or localized disease with unfavorable markers, such as MYCN amplification.

### Statistical analysis

To determine robust features, half of the patients were selected randomly and segmented independently by another nuclear medicine physician with 6 years of PET/CT experience. We evaluated the reproducibility of features using a two-way random, absolute agreement intraclass correlation coefficient (ICC). Using the lower bounds of the 95% confidence interval (CI) of the ICC value (ICC_lb95%_) (18), the reproducibility of each feature was categorized as follows: poor, ICC_lb95%_ <0.50; moderate, ICC_lb95%_ of 0.50–0.75; good, ICC_lb95%_ of 0.75–0.90; and excellent, ICC_lb95%_ ≥0.90. Robust features with good or excellent reproducibility were qualified for further analysis.

The Mann–Whitney *U* test and chi-squared test were used for comparing variables between groups. The Benjamini–Hochberg stepwise method was performed to control the false discovery rate and adjusted *p*-values were calculated. Correlations among the parameters were determined by the Pearson and Spearman rank correlation. To avoid redundancy, factors with poorer predictive validity in the pairs of indices that showed correlation coefficient (*r*) ≥0.8 were omitted (19, 20). Logistic regression analyses with forward selection were performed to evaluate the relationship between imaging indices and MYCN amplification. Then, the entire cohort was randomly split into a training set (*n* = 77) and a test set (*n* = 35). Prognostic factors were identified by univariate and multivariable Cox regression analyses in the training set and then validated in the test set. Receiver-operating characteristic curve (ROC) analyses and the Youden index were used to determine the optimal cutoff values. Survival estimates were evaluated by the Kaplan–Meier analysis and log-rank test. All statistical analyses were performed using SPSS 25.0 (IBM, Chicago, IL, USA), except that the adjusted *p*-values were obtained on R software (Version 4.0.3, http://www.r-project.org/). A two-sided *p*-value <0.05 was considered statistically significant.

## Results

### Patient characteristics

As a result, a total of 112 children were identified. The patient characteristics are summarized in **Table 1**. There were 39 girls (median age 34 months, range 1–153 months) and 73 boys (median age 36 months, range 2–150 months). Ninety patients had neuroblastoma and 22 had ganglioneuroblastoma (GNB). Most of them presented disseminated disease (2 with stage 4S, 79 with stage 4). With a median follow-up of 33 months, 51 disease relapse/progression and 34 deaths occurred. The 3-year EFS rate was 47%.

**Table 1:** Patient characteristics.

Characteristics	Total (*n* = 112)	Training set (*n* = 77)	Test set (*n* = 35)	*p-*value
	No. (%)	No. (%)	No. (%)	
Median age (months)	37 ± 26	37 ± 28	37 ± 22	0.615
≥18 months	84 (75%)	58 (75%)	26 (74%)	0.906
Sex				0.438
Female	39 (35%)	25 (32%)	14 (40%)	
Male	73 (65%)	52 (68%)	21 (60%)	
Pathology				0.081
GNB intermixed/well-differentiated	16 (14%)	8 (10%)	8 (23%)	
GNB nodular, neuroblastoma	96 (86%)	69 (90%)	27 (77%)	
MYCN (*n* = 90)				0.320
Non-amplified	70 (78%)	48/64 (75%)	22/26 (85%)	
Amplified	20 (22%)	16/64 (25%)	4/26 (15%)	
Location				0.352
Abdominal and pelvic	92 (82%)	65 (84%)	27 (77%)	
Others	20 (18%)	12 (16%)	8 (23%)	
Stage				0.889
1, 2, 3, 4S	33 (29%)	23 (30%)	10 (29%)	
4	79 (71%)	54 (70%)	25 (71%)	
Risk stratification				0.910
Low	7 (6%)	5 (6%)	2 (6%)	
Intermediate	26 (23%)	17 (22%)	9 (26%)	
High	79 (71%)	55 (71%)	24 (69%)	
Laboratory tests				
Ferritin ≥92 ng/ml^a^	63/96 (66%)	45/64 (70%)	18/32 (56%)	0.171
LDH ≥587 U/L^a^	51/100 (51%)	36/66 (55%)	15/34 (44%)	0.323
Metabolic parameters				
SUVmax	6.3 ± 3.5	6.1 ± 3.2	6.7 ± 4.3	0.488
SUVpeak	4.6 ± 2.4	4.5 ± 2.1	4.8 ± 3.1	0.713
Endpoints				
Progression	51 (46%)	33 (43%)	18 (51%)	0.399
Death	34 (30%)	21 (27%)	13 (37%)	0.292

LDH, lactate dehydrogenase; GNB, ganglioneuroblastoma.

a Cutoff values for ferritin and LDH were set according to INRG (2).

All the patients had an FDG-avid primary tumor with a median SUVmax of 5.8 (range 1.6–26.5). Seven tumors had SUVmax lower than 2.5 (1.6–2.4), all of which were higher than the liver background. High-risk neuroblastoma showed significantly higher FDG uptake (SUVmax and SUVpeak, *p* < 0.001) and volumetric values (MTV, TLG, and TLG41%, all *p* < 0.001; MTV41%, *p* = 0.040) than those with non-high-risk disease (**Figure S1**).

### Imaging feature selection

Sixty-nine imaging features were obtained per VOI. The steps used to reduce feature dimension are summarized in **Table S1**. The ICC revealed that most of the imaging features could be reproduced well (**Table S2**). Fifty-one out of the 59 feature pairs had ICC_lb95%_ ≥0.75 (excellent reproducibility in 39 and good reproducibility in 12) and were qualified for subsequent analyses.

### Imaging model for predicting MYCN amplification

MYCN status was available in 90 patients and was amplified in 20 patients. The majority of imaging features (44/51) were significantly different between the MYCN-amplified and the non-amplified groups (**Table S3**). After false discovery correction, 34 remained statistically significant. For example, FDG uptake was significantly higher in the MYCN-amplified tumor (SUVmax: 7.9, 95% CI: 6.7–9.9 vs. 5.1, 95% CI: 4.9–6.6, *p* < 0.001, adjusted *p* = 0.005).

**Figure 2:**
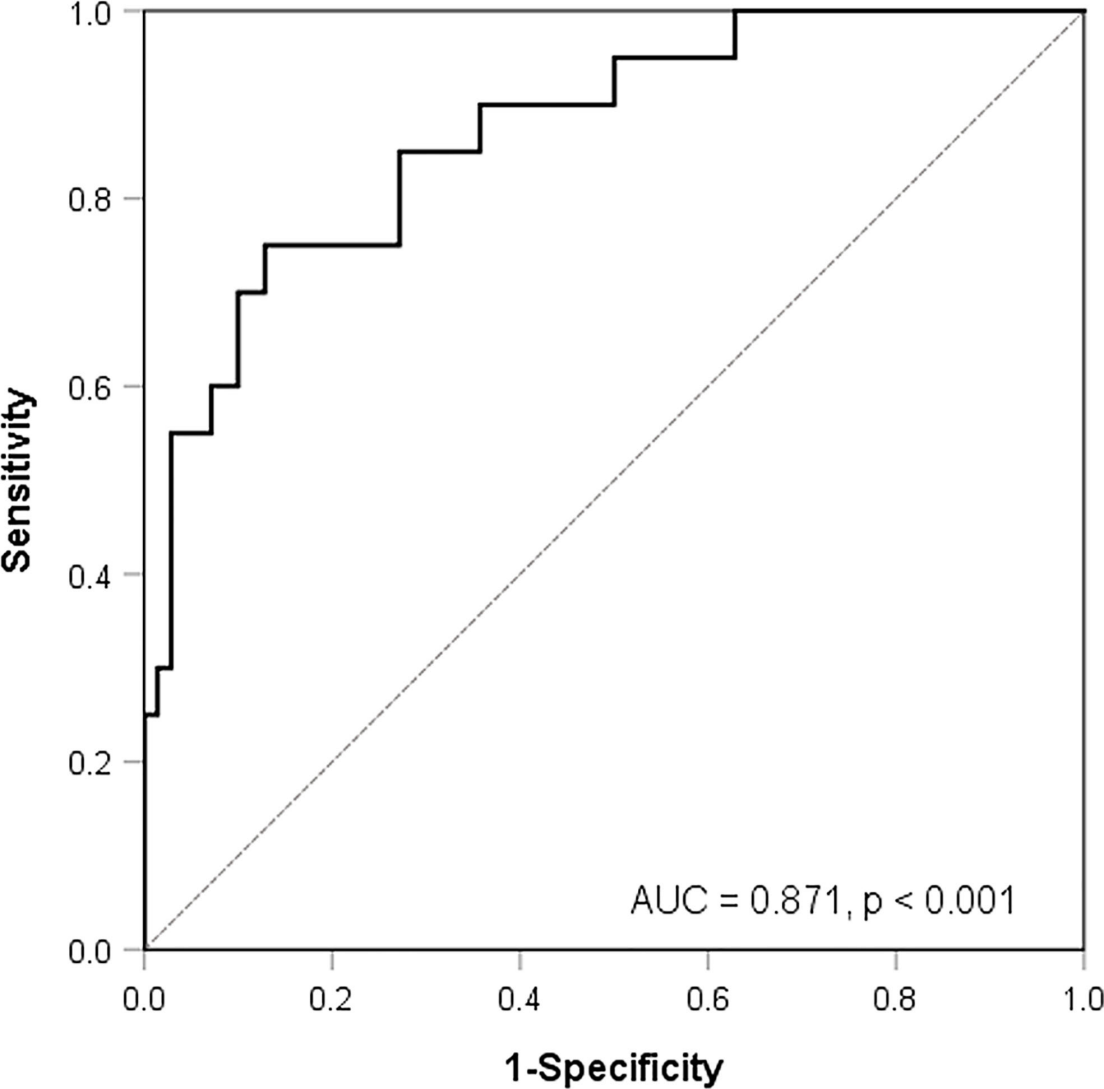
Receiver-operating characteristic curve analysis for the prediction of MYCN amplification according to a model composed of two texture features.

The ROC analysis showed that all of the above 34 features had AUCs higher than 0.7 to predict MYCN amplification. Histogram_Kurtosis, which reflects the shape of the histogram distribution relative to a normal distribution, yielded the highest AUC of 0.853 (*p* < 0.001). After multicollinearity reduction, nine features were entered into multivariate logistic regression analysis. A radiomic model composed of two features [Histogram_Kurtosis and gray-level non-uniformity from gray-level zone length matrix (GLZLM_GLNU), which reflects the non-uniformity of the gray levels of the homogeneous zones in 3D] was built subsequently, resulting in an AUC of 0.871 (**Figure 2**, *p* < 0.001) with the following equation:

Predicted probability = EXP (−0.287 − 0.228 × Histogram_Kurtosis + 0.021 × GLZLM_GLNU)/(1 + EXP (−0.287 − 0.228 × Histogram_Kurtosis + 0.021 × GLZLM_GLNU).

### Development of rad-risk to predict EFS

The distribution of key variables including age, stage, MYCN, and conventional metabolic parameters was similar between the training and test sets (**Table 1**). In the training set, univariate Cox regression analysis revealed that 14 first-order and 8 second-order indices correlated with EFS (*p* < 0.05). After feature dimension reduction, two first-order indices, namely, SUVmax and Histogram_Entropy reflecting the randomness of the voxel distribution, were retained (**Table 2**). Three second-order indices retained were as follows: one from the gray-level co-occurrence matrix (GLCM): GLCM_energy, which reflects the uniformity of gray-level voxel pairs; two from the gray-level run-length matrix (GLRLM), namely, the gray-level non-uniformity (GLRLM_GLNU), which measures the non-uniformity of the gray levels, and run-length non-uniformity (GLRLM_RLNU), which quantifies the non-uniformity of the length of the homogeneous runs. The AUCs for SUVmax, Histogram_Entropy, GLCM_energy, GLRLM_GLNU, and GLRLM_RLNU to predict progression were 0.611, 0.666, 0.645, 0.689, and 0.733, respectively. Multivariate Cox regression analyses revealed that GLRLM_RLNU with a cutoff value of 1,828 and Histogram_Entropy with a cutoff value of 3.3 outperformed other imaging indices and were significant to predict events. In addition, imaging features extraction was performed in the high-risk group separately, and the results are presented in the **Supplementary Materials** (**Supplementary Data** and **Figure S2**).

**Table 2:** Univariate Cox regression analyses for event-free survival.

Variables	Training set (*n* = 77)			Test set (*n* = 35)		
	HR	95% CI	*p*-value	HR	95% CI	*p*-value
Clinicopathological factors						
Age ≥18 months	13.2	1.8–96.8	0.011	2.1	0.6–7.3	0.246
Stage 4 vs. 1, 2, 3, 4S	4.5	1.6–12.9	0.005	2.5	0.6–10.9	0.224
MYCN amplification^a^	2.8	1.2–6.4	0.014	0.5	0.1–4.1	0.527
LDH ≥587^b^	3.0	1.3–6.9	0.008	3.3	1.3–8.6	0.015
Ferritin ≥92^c^	3.0	1.1–7.8	0.026	1.9	0.7–5.3	0.246
First-order imaging indices						
SUVmax ≥5.5	3.2	1.5–6.7	0.003	2.9	1.0–8.1	0.049
Histogram_Entropy ≥3.3	3.8	1.8–7.9	<0.001	3.5	1.1–10.6	0.029
Second-order imaging indices						
GLCM_Energy ≤0.02	3.5	1.7–7.1	0.001	2.1	0.8–5.6	0.146
GLRLM_RLNU ≥1,828	5.1	2.3–11.3	<0.001	5.7	2.0–16.4	0.001
GLRLM_GLNU ≥575	2.9	1.5–5.8	0.002	2.1	0.6–6.6	0.224

CI, confidence interval; HR, hazard ratio; GLCM, gray level co-occurrence matrix; GLNU, gray-level non-uniformity; GLRLM, gray-level run-length matrix; rad-risk, radiomic risk; RLNU, run-length non-uniformity.

a MYCN amplification status was available in 64 patients in the training set and 26 in the test set.

b LDH was available in 66 patients in the training set and 34 in the test set.

c Ferritin was available in 64 patients in the training set and 32 in the test set.

**Figure 3:**
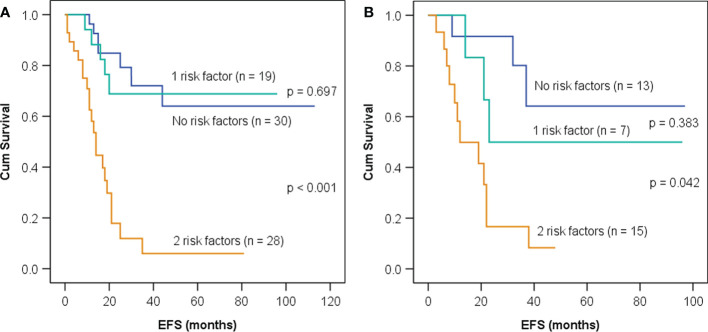
Kaplan–Meier event-free survival (EFS) curves in children with neuroblastoma having neither, one, or both imaging risk factors—GLRLM_RLNU ≥1,828 and Histogram_Entropy ≥3.3—in the training set **(A)** and the test set **(B)**.

Then, patients in the training set and the test set were divided into three groups according to whether GLRLM_RLNU ≥1,828 and Histogram_Entropy ≥3.3: patients with neither of these two risk factors, those with either one of the factors, and those with both. Patients with neither or either one of these factors demonstrated similar survival curves both in the training set and test set (**Figure 3A**, *p* = 0.697; **Figure 3B**, *p* = 0.383) and, thus, were combined and categorized as low rad-risk. Patients with both factors had a significantly worse prognosis (training set, HR: 6.4, 95% CI: 3.1–13.2, *p* < 0.001; test set, HR: 5.0, 95% CI: 1.8–13.6, *p* = 0.002) and were categorized as high rad-risk. The 3-year EFS of low vs. high rad-risk was 71% vs. 6% in the training set and 69% vs. 17% in the test set, respectively (both *p* < 0.001).

### Multivariate analysis

Clinicopathological factors including age, stage, MYCN, LDH, and ferritin significantly correlated with EFS in the training set (**Table 2**). As MYCN status, LDH, and ferritin were unavailable in several patients, we firstly integrated rad-risk with age and stage into the multivariate analysis. After adjustment for clinical covariates (**Table 3**), rad-risk obtained independent significance with HR of 4.3 (95% CI: 2.0–9.1, *p* < 0.001), while age showed marginal significance (HR: 6.8, 95% CI: 0.9–52.5, *p* = 0.066). After incorporating MYCN into the model, only rad-risk remained significant (HR: 8.8, 95% CI: 3.7–21.0, *p* < 0.001). Furthermore, we integrated LDH and ferritin into the multivariate analyses separately or together, and rad-risk was the only factor that retained significance.

**Table 3:** Multivariate Cox regression analyses for event-free survival.

Models	Training set			Test set		
	HR	95% CI	*p-*value	HR	95% CI	*p-*value
Multivariate model 1^a^	*n* = 77			*n* = 35		
Age ≥18 months	6.8	0.9–52.5	0.066	/	/	/
High rad-risk	4.3	2.0–9.1	<0.001	5.0	1.8–13.6	0.002
Multivariate model 2^b^	*n* = 64			*n* = 26		
High rad-risk	8.8	3.7–21.0	<0.001	6.7	1.7–26.0	0.007
Multivariate model 3^c^	*n* = 66			*n* = 34		
Age ≥18 months	6.5	0.8–49.9	0.074	/	/	/
LDH ≥587 U/L	/	/	/	3.2	1.1–8.7	0.026
High rad-risk	4.4	2.0–9.6	<0.001	4.8	1.7–13.6	0.003
Multivariate model 4^d^	*n* = 64			*n* = 32		
Age ≥18 months	7.0	0.9–54.1	0.062	/	/	/
High rad-risk	3.9	1.8–8.5	<0.001	4.4	1.6–12.2	0.004
Multivariate model 5^e^	*n* = 51			*n* = 25		
High rad-risk	8.3	3.2–21.4	<0.001	12.9	2.6–63.6	0.002

a Multivariate model 1 includes age, stage, and rad-risk (n = 112).

b Multivariate model 2 includes age, stage, MYCN, and rad-risk (n = 90).

c Multivariate model 3 includes age, stage, LDH, and rad-risk (n = 100).

d Multivariate model 4 includes age, stage, ferritin, and rad-risk (n = 96).

e Multivariate model 5 includes age, stage, MYCH, LDH, ferritin, and rad-risk (n = 76).

Similarly, after adjusting for clinicopathological variables separately or together in the multivariate analysis, high rad-risk was confirmed to be the most significant factor to predict EFS in the test set (**Table 3**).

### Refinement of risk stratification in neuroblastoma

None of the seven patients with clinical low-risk diseases had a high rad-risk, and only 2 of the 26 patients with clinical intermediate-risk diseases had a high rad-risk, indicating that the majority of patients with clinical non-high-risk had a relatively homogeneous tumor. Due to limited cases with a high rad-risk in the clinical non-high-risk group, the significance of rad-risk in the risk substratification in this group could not be statistically analyzed.

Seventy-nine patients had high-risk neuroblastoma: 55 patients in the training set and 24 in the test set. We further evaluated whether adding rad-risk could refine risk stratification and compared it with volumetric indices, including MTV, MTV41%, TLG, and TLG41%. In the training set, ROC analyses were performed (**Figure S3**) and optimal cutoff values were determined to be 120 ml for MTV, 65 ml for MTV41%, 426 g for TLG, and 141 g for TLG41%, respectively.

**Figure 4:**
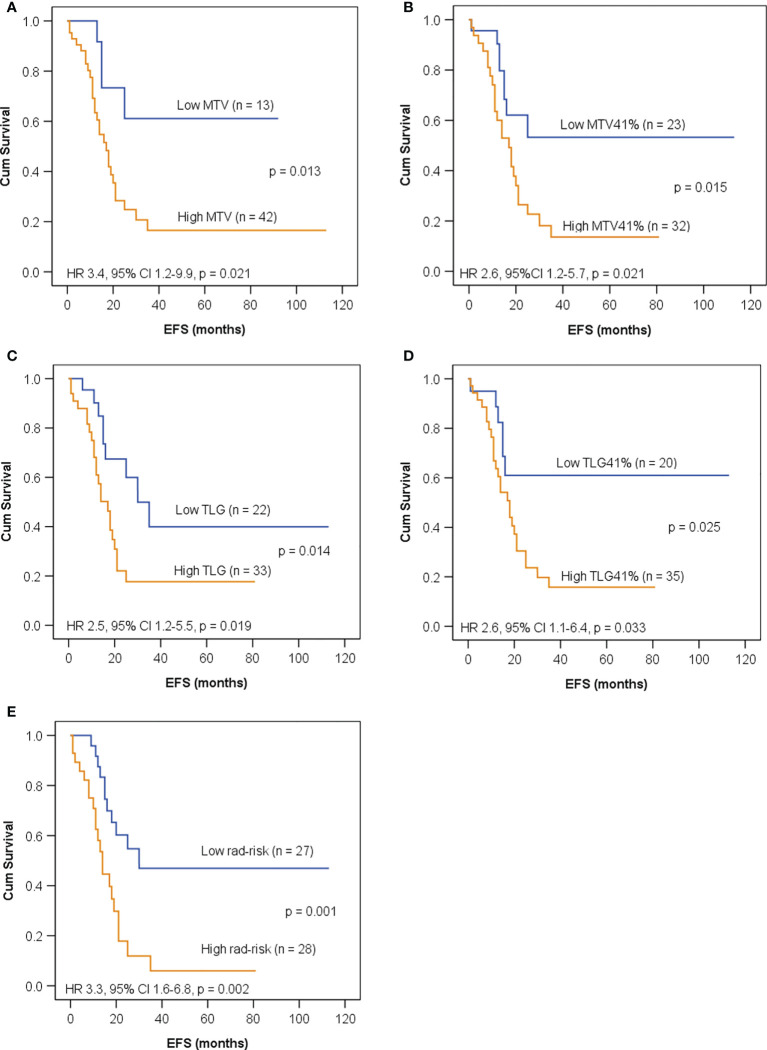
Kaplan–Meier curves for EFS in children with high-risk neuroblastoma in the training set according to **(A)** metabolic tumor volume (MTV) with a cutoff value of 120 ml; **(B)** MTV41% with a cutoff value of 65 ml; **(C)** total lesion glycolysis (TLG) with a cutoff value of 426 g; **(D)** TLG41% with a cutoff value of 141 g; **(E)** rad-risk.

**Figure 5:**
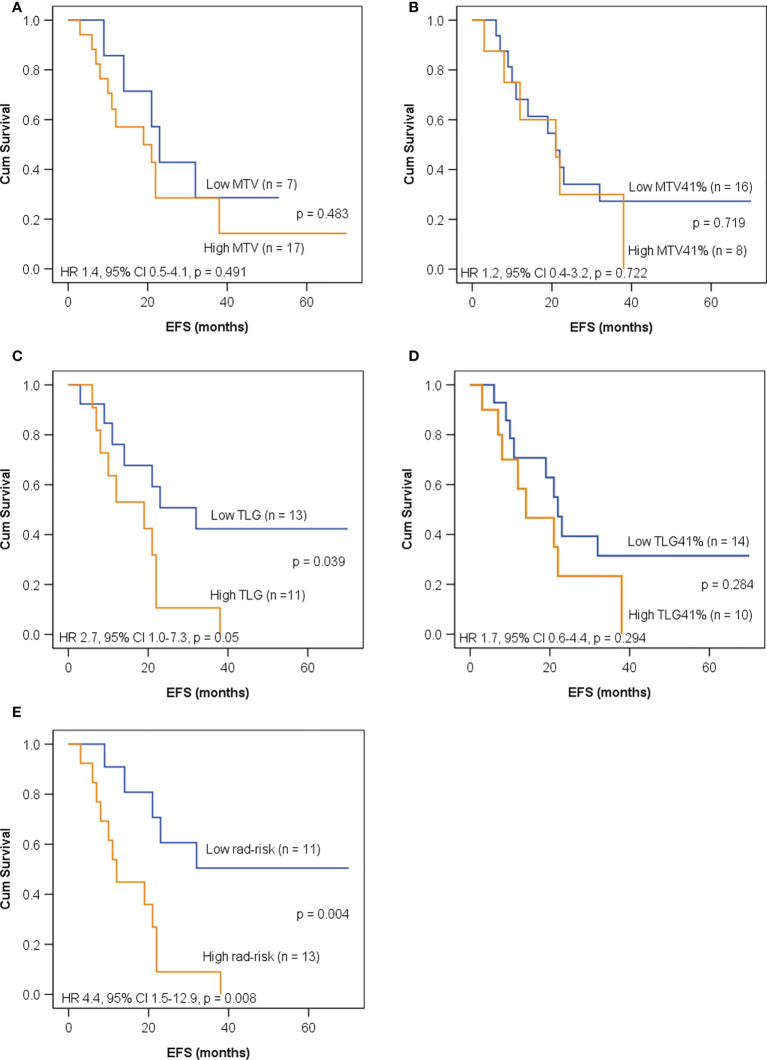
Kaplan–Meier curves for EFS in children with high-risk neuroblastoma in the test set according to **(A)** MTV with a cutoff value of 120 ml; **(B)** MTV41% with a cutoff value of 65 ml; **(C)** TLG with a cutoff value of 426 g; **(D)** TLG41% with a cutoff value of 141 g; **(E)** rad-risk.

**Figure 6:**
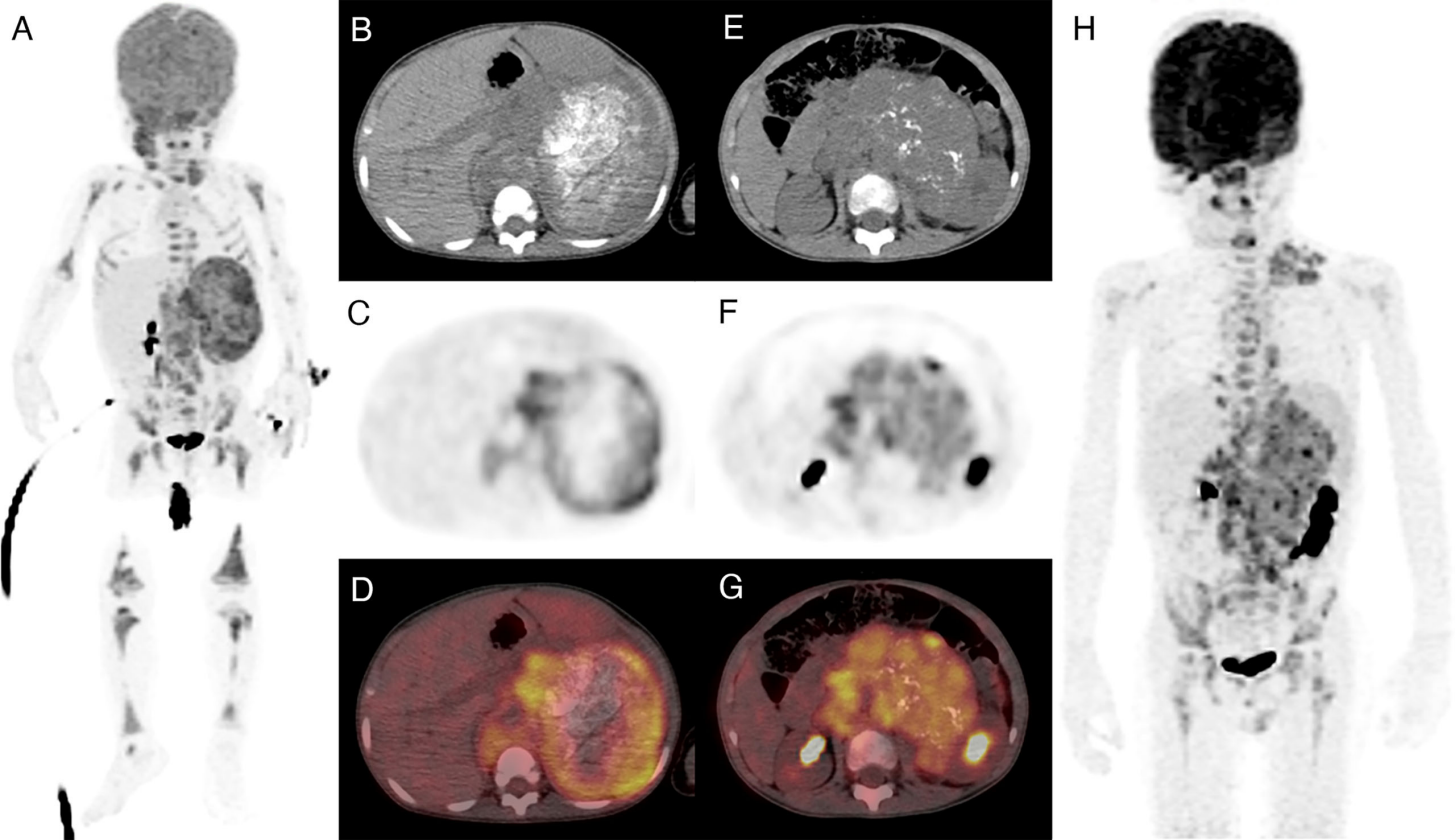
Two patients with a high-risk neuroblastoma and high or low intratumor heterogeneity. Both patients had amplified MYCN and stage 4 diseases. **(A–D)** A 20-month-old girl with a highly heterogeneous FDG uptake in the primary tumor (high rad-risk). She progressed 21 months after diagnosis. **(E–H)** A 5-year-old boy with a relatively homogeneous FDG uptake (low rad-risk). The patient remained recurrence free within 5 years of follow-up.

As shown in **Figure 4**, all of the five imaging indices significantly correlated with EFS in the training set. The 3-year EFS for patients with high vs. low MTV, MTV41%, TLG, and TLG41% were 17% vs. 61% (*p* = 0.013), 14% vs. 53% (*p* = 0.015), 18% vs. 40% (*p* = 0.014), and 16% vs. 61% (*p* = 0.025), respectively. However, the volumetric features failed to retain significance in the test set, except TLG (**Figure 5**). Rad-risk yielded the best performance to distinguish high-risk patients with different outcomes, with a 3-year EFS of 6% vs. 47% (*p* = 0.001, **Figure 4E**) in the training set and 9% vs. 51% (*p* = 0.004, **Figure 5E**) in the test set. High rad-risk was associated with a 2.3–3.4 times higher risk of progression (HR: 3.3, 95% CI: 1.6–6.8, *p* = 0.002 in the training set; HR: 4.4, 95% CI: 1.5–12.9, *p* = 0.008 in the test set). Two patients with high-risk neuroblastoma and a high or low rad-risk are presented in **Figure 6**.

## Discussion

The substratification of high-risk neuroblastoma is challenging, and new predictive biomarkers are warranted for better patient selection. In this study, we confirmed that PET-based intratumor heterogeneity independently correlated with EFS in neuroblastoma both in the training set and the test set. It further improved the risk stratification in high-risk neuroblastoma, with a 3-year EFS of 6%–9% for the highly heterogeneous tumors compared to 47%–51% for the relatively homogeneous ones.

Radiomics, extracting quantitative features from medical images, has rapidly evolved throughout these years. Compared to histological biopsy only capturing a small proportion of tumor tissue that could underestimate the mutational burden (21), a great advantage of radiomics is its ability to visualize the characteristics of the whole tumor non-invasively. It fully depicts spatial intratumor heterogeneity, which has been associated with poor prognosis. Studies showed that radiomic features in PET images correlated with heterogeneity at the cellular and genomic levels and had significant prognostic value in various malignancies (22–24). On the other hand, tumor necrosis results from increased tumor size, intratumor hypoxia, and nutrient deprivation. Both the presence and the extent of necrosis correlated with poor prognosis (25, 26). A necrotic core appears as non-FDG-avid area within the tumor. To investigate the spatial voxel relationships inside the entire tumor, the current study examined the VOI covering the whole mass (including the necrotic region) instead of putting a threshold of SUVmax on VOI segmentation. Our results partly confirmed previous studies that texture features significantly correlated with tumor size or volume (27, 28). We found four second-order indices reflecting tumor heterogeneity, including GLRLM_RLNU, highly correlated with volumetric indices. The latter is usually considered a reflection of tumor burden, while texture features correlate with tumor heterogeneity. A larger tumor results in a higher level of intratumor hypoxia and necrosis and leads to higher spatial complexity and heterogeneity (27). Hatt et al. found that radiomic heterogeneity quantification provided valuable complementary information for large tumors (>10 cm^3^) (27). In our study, only one patient had a tumor volume less than 10 ml. The median volume for our whole cohort was 160 ml.

MYCN amplification is the most common genomic alteration in neuroblastoma, occurring in approximately 20% of the patients (29). It is highly associated with advanced stage and poor prognosis; thus, it has been incorporated into the mostly used neuroblastoma protocol. Radiomic models derived from contrast-enhanced CT have been shown to accurately predict MYCN amplification (30–32). Wu et al. (31) suggested that three-phase CT had a higher value than non-contrast CT scan, which could be explained by tumor angiogenesis promoted by MYCN amplification. Different from the density heterogeneous and vascular structure complexity depicted by CT scans, PET imaging semi-quantifies the glucose consumption of tumor parenchyma and reflects the uneven spatial distribution of cellular metabolism, hypoxia, necrosis, and proliferation. In our study, MYCN amplification occurred in 22% of the patients. Two patients had divergent MYCN results, potentially resulting from the heterogeneity of the tumor or underestimation of the mutational burden by biopsy bias. In line with a prior study by Sung et al. (33), we found that SUVmax and TLG had the potential to predict MYCN with AUCs of 0.771 and 0.776, respectively. However, histogram metrics and several second-order indices showed superior performance. Consequently, a radiomic model containing two PET features, namely, Histogram_Kurtosis and GLZLM_GLNU, was built and showed the strongest predictive power with an AUC of 0.871. Histogram_Kurtosis reflects the shape of the histogram distribution relative to a normal distribution. GLZLM_GLNU reflects the non-uniformity of the gray levels of the homogeneous zones in 3D. These two features have been proven to be promising parameters as biomarkers of tumor heterogeneity in various malignancies (34–36). A higher GLZLM_GLNU and a lower Histogram_Kurtosis, which indicate higher spatial heterogeneity, correlated with a higher possibility of MYCN amplification. Recently, Qian et al. reported that the radiomic signature containing both PET and CT features had a good ability to predict MYCN amplification (37). However, the majority of the features were obtained from wavelet transformed images, which decompose an image by using spatially oriented frequency filters but require intensive computation and may suffer from low reproducibility (38). Despite the methodology differences, we both showed that a high intratumor heterogeneity was associated with MYCN amplification. Since neuroblastoma is remarkably heterogeneous, which might require at least two solid tumor areas to provide a more accurate genomic diagnosis (39), texture features fully portraying the entire tumor might provide important complementary information about molecular profiling.

Our second step was to evaluate whether intratumor heterogeneity could provide prognostic information in pretreatment neuroblastoma. A recent study reported that high intratumor metabolic heterogeneity on ^18^F-FDG PET/CT was a strong prognostic factor in 38 children with newly diagnosed neuroblastoma (40), and it was the first report identifying metabolic heterogeneity as a prognostic biomarker of neuroblastoma. The authors used the area under the curve of the cumulative SUV-volume histograms (AUC-CSHs), which is a histogram-based first-order feature that describes the percentage of total tumor volume above the percent threshold of SUVmax, as an intratumor heterogeneity index. Lower AUC-CSH indicated higher heterogeneity of the tumor and poorer outcomes. Although the histogram analysis appears promising and simple, the major pitfalls of the histogram analysis are the lack of information on the spatial organization of tumors and that it is not straightforward which might lead to errors (34, 41). In another recently published study of 18 children with high-risk neuroblastoma, Fiz et al. demonstrated that intratumor heterogeneity on 18fluorine-dihydroxyphenylalanine (18F-DOPA) PET/CT was closely associated with metastatic burden and had certain prognostic value (42). In the current study, we further expanded that intratumor heterogeneity was a prognostic biomarker in neuroblastoma, with a much larger cohort and a higher order of texture analysis, which further improves quantitative histogram approaches by introducing the spatial dimension. Multivariate analysis identified GLRLM_RLNU and Histogram_Entropy as the independently significant predictors for EFS. GLRLM_RLNU gives the size of homogeneous runs for each gray level. A similar run length results in low values of GLRLM_RLNU. On the contrary, a high value is indicative of heterogeneity. Studies have reported that GLRLM_RLNU extracted from PET had the potential for predicting treatment response and prognosis (43, 44). On the other hand, Histogram_Entropy measures the randomness of voxel distribution and has been established as an important biomarker reflecting heterogeneity in various MRI and PET studies (43, 45). In accordance with previous studies (40, 42), we found that high rad-risk, defined as patients with both a high GLRLM_RLNU and a high Histogram_Entropy, indicating a high intratumor heterogeneity, was the most significant independent factor for EFS after adjusting for clinicopathological factors.

To further evaluate the ability of rad-risk in the refinement of risk stratification, we incorporated rad-risk into the existing risk stratification schema and compared it to volumetric indices. The results showed that the majority of patients with clinical non-high risk had a low rad-risk, indicating a relatively homogeneous tumor. Among high-risk neuroblastoma, rad-risk effectively distinguished patients with distinct outcomes both in the training and test sets. In addition, despite that intratumor heterogeneity highly correlated with MTV and TLG, rad-risk outperformed the volumetric indices and showed the highest ability to predict the outcome. These findings indicate that PET-based intratumor heterogeneity might have independent prognostic information, which may help substratify neuroblastoma patients for more refined risk-adapted treatment approaches in the future.

The limitations of this study are as follows: first, this is a retrospective study with a relatively small sample size in a single center. Second, ^123^I-mIBG scans were not performed in our cohort, since ^123^I-MIBG is not yet available in our country. The disadvantages of the ^123^I-mIBG scan, including limited spatial resolution and lower sensitivity in soft tissue lesions or small lesions, limit its value in radiomic analysis in neuroblastoma. Future efforts in PET-based texture features using novel radiopharmaceuticals such as ^18^F-fluorometaguanidine and ^124^I-mIBG might yield important predictive or prognostic information. Third, this study evaluated the features of primary tumor and captured less information outside the primary site, such as metastatic lesions or metastatic burden, which could be of important prognostic value. An additional limitation is that no separate cohort was used for validation regarding the prediction of MYCN amplification due to the limited number of patients with amplified MYCN. A large cohort with external validation should be warranted in the future.

## Conclusions

In summary, PET-based intratumor heterogeneity could serve as a powerful and non-invasive approach to predict MYCN amplification and survival outcome in newly diagnosed neuroblastoma, providing a potential approach to refine the risk stratification in children with high-risk diseases. Further validation with a larger cohort is required.

## Data availability statement

The original contributions presented in the study are included in the article/**Supplementary Material**. Further inquiries can be directed to the corresponding authors.

## Ethics statement

This retrospective study was approved by the Ethics Committee of Xin Hua Hospital Affiliated to Shanghai Jiao Tong University School of Medicine and the requirement for informed consent was waived.

## Author contributions

ChL, HW, and SC contributed to the conception and design of the study. ChL and SC organized the database. ChL and SC performed the statistical analysis. CaL, SW, YY, FF, and HF performed the data analysis and interpretation. ChL and SC wrote the first draft of the manuscript. CaL, SW, YY, FF, and HF wrote sections of the manuscript. ChL, HW, and SC edited the manuscript. All authors contributed to manuscript revision, read, and approved the submitted version.

## Funding

This study has been supported by the National Natural Science Funds (81801731 and 81901775).

## Conflict of interest

The authors declare that the research was conducted in the absence of any commercial or financial relationships that could be construed as a potential conflict of interest.

## Publisher’s note

All claims expressed in this article are solely those of the authors and do not necessarily represent those of their affiliated organizations, or those of the publisher, the editors and the reviewers. Any product that may be evaluated in this article, or claim that may be made by its manufacturer, is not guaranteed or endorsed by the publisher.
