# Supplementary material for: Improved risk stratification by PET-based intratumor heterogeneity in children with high-risk neuroblastoma
Source: Front Oncol. 2022 Oct 24;12:896593. doi: 10.3389/fonc.2022.896593 (PMC9637983; doi:10.3389/fonc.2022.896593)
Supplement: Supplementary file 1 [file DataSheet_1.docx]

Supplementary Material

# Supplementary Data

# Development of imaging features to refine risk stratification in high-risk neuroblastoma

# In addition, we have tried to extract imaging features in high-risk patients separately. Univariate Cox regression analysis revealed that 2 first-order [Shape_Volume(ml) and Shape_Surface(mm2)] and 2 second-order indices (GLRLM_RLNU and GLZLM_GLNU) were significantly associated with EFS (p < 0.05). However, GLRLM_RLNU highly correlated with the other 3 features (r = 0.917 with Shape_Volume(ml), r = 0.883 with Shape_Surface(mm2), and r = 0.926 with GLZLM_GLNU). To avoid redundancy, the 3 features with lower predictive values were omitted. The optimal cutoff value of 1828 for GLRLM_RLNU was determined by ROC analysis and the Youden index. Survival analysis revealed that GLRLM_RLNU alone predicted events efficiently both in the training and test sets (Supplementary Figure 3), less powerful than rad-risk, however, which could be explained by underpowering of Histogram_Entropy due to the smaller sample size of the high-risk group than the entire cohort.

# Supplementary Figures

**
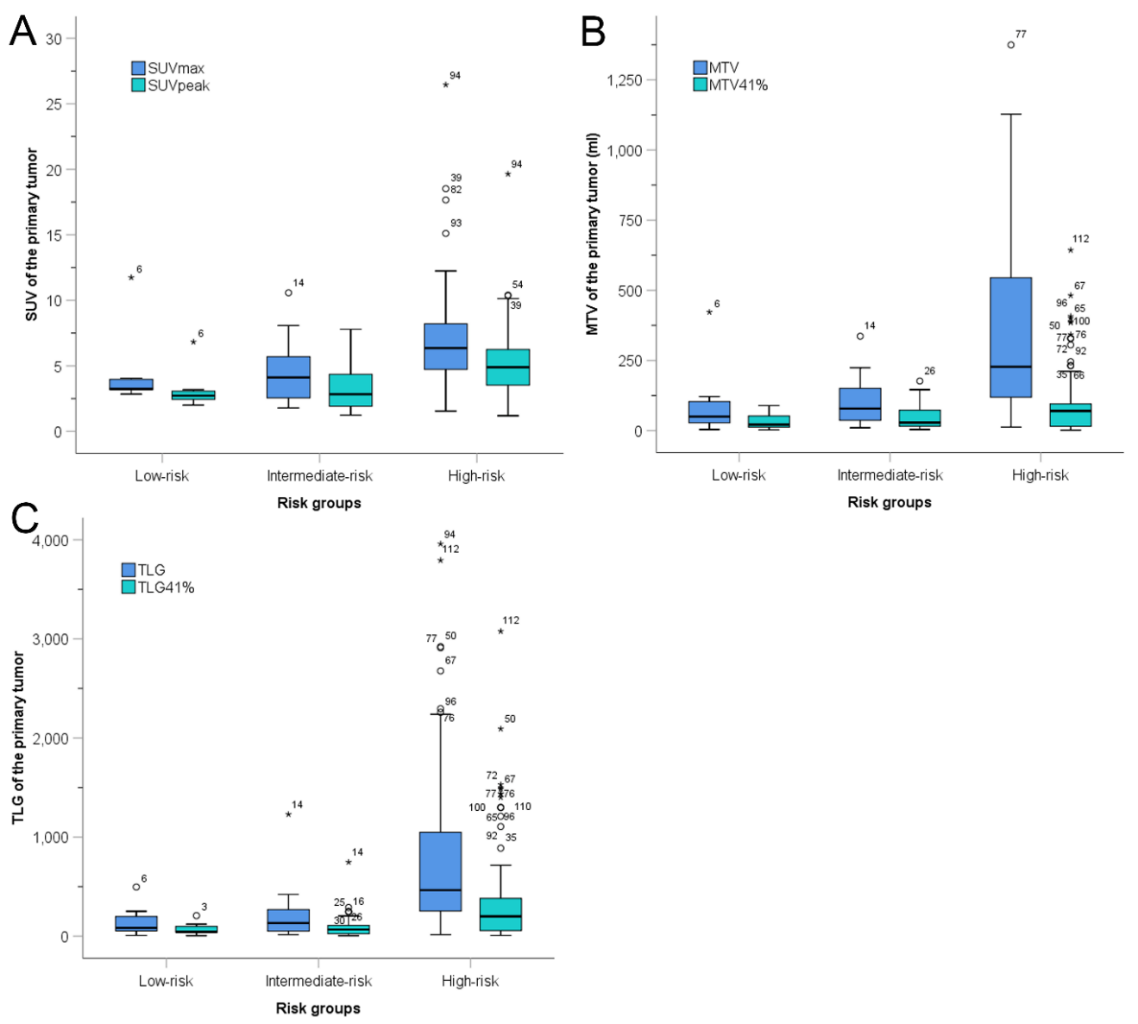
**

**Supplementary Figure 1.** Boxplots of PET-based metabolic and volumetric parameters of the primary tumors in children with neuroblastoma according to risk classification: low-risk (n = 7), intermediate-risk (n = 26), and high-risk (n = 79): (A) SUVmax and SUVpeak; (B) metabolic tumor volume (MTV) and MTV41%; (C) total lesion glycolysis (TLG) and TLG41%.

*
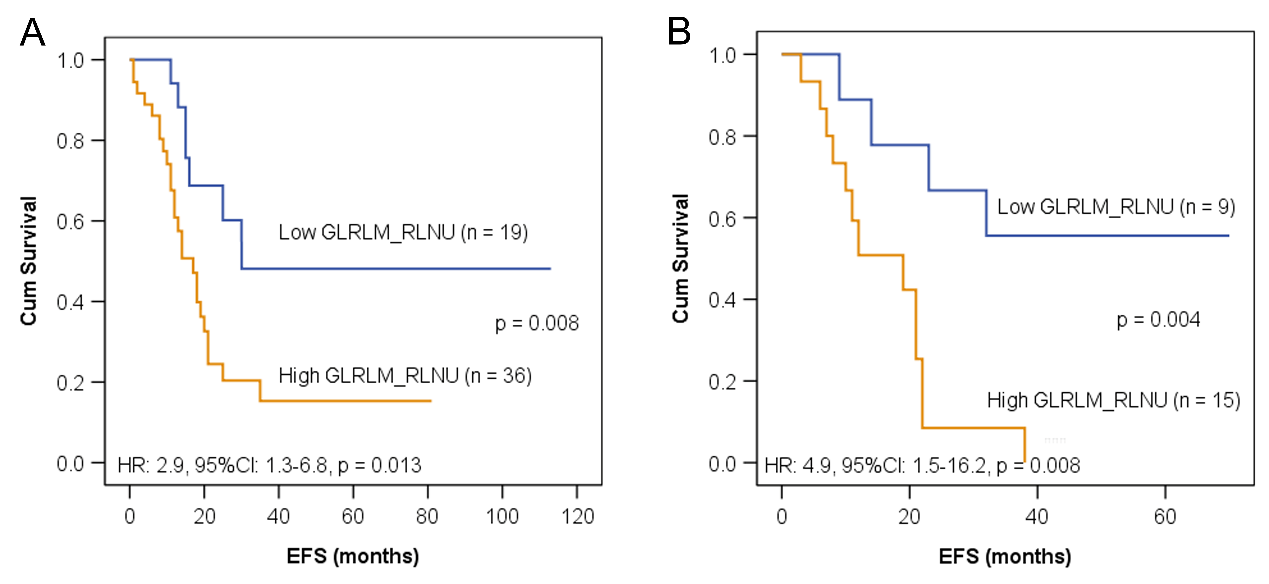
*

**Supplementary Figure 2.** Kaplan-Meier curves for EFS in children with high-risk neuroblastoma and low or high GLRLM_RLNU with a cutoff value of 1828 in the training set (A) and the test set (B). 3-year EFS for patients with high vs. low rad-risk were 48% vs. 15% (p = 0.008) in the training set and 56% vs. 9% (p = 0.004) in the test set, respectively.


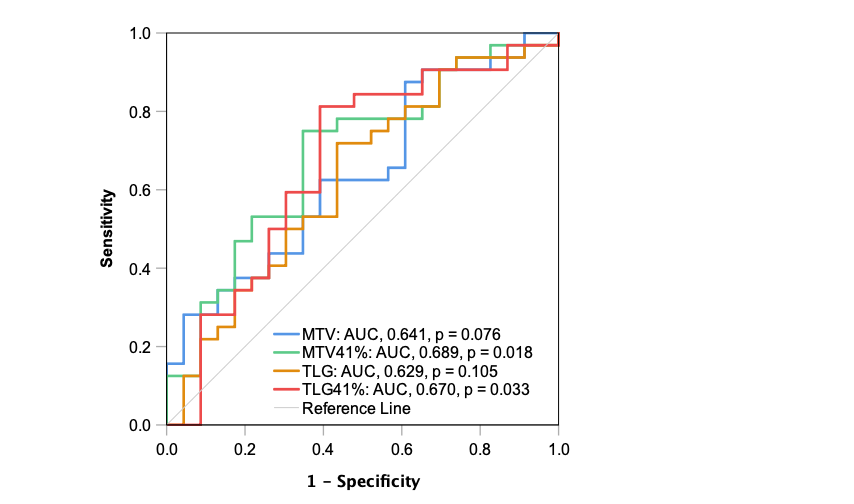


**Supplementary Figure 3.** Receiver-operating characteristic curve analyses for prediction of events according to MTV, MTV41%, TLG, and TLG41% in the patients with high-risk disease in the training set (n = 55). AUC: area under the curve; MTV: metabolic tumor volume; MTV41%: MTV with a threshold of 41% of SUVmax; TLG: total lesion glycolysis; TLG41%: TLG with a threshold of 41% of SUVmax.

# Supplementary Tables

**Supplementary Table 1. Feature selection steps**

| **Features selection step** | **First-order features** | | | **Second-order features** | | | | **Total** |
| --- | --- | --- | --- | --- | --- | --- | --- | --- |
|  | **Conventional** | **Histogram** | **Shape** | **GLCM** | **GLRLM** | **NGLDM** | **GLZLM** |  |
| Before selection | 26 | 6 | 5 | 7 | 11 | 3 | 11 | 69 |
| Exact linear (*r*=1/-1) | 19 | 4 | 5 | 6 | 11 | 3 | 11 | 59 |
| Reproducibility (ICC_lb95%_ ≥0.75)^a^ | 17 | 4 | 3 | 5 | 11 | 2 | 9 | 51 |
| MYCN prediction |  |  |  |  |  |  |  |  |
| Significance | 14 | 4 | 3 | 5 | 11 | 1 | 6 | 44 |
| False discovery rate | 10 | 4 | 2 | 5 | 6 | 1 | 6 | 34 |
| Model | 0 | 1 | 0 | 0 | 0 | 0 | 1 | 2 |
| Event-free survival |  |  |  |  |  |  |  |  |
| Univariate | 7 | 4 | 3 | 2 | 2 | 0 | 4 | 22 |
| Redundancy (*r* ≥0.8) | 1 | 1 | 0 | 1 | 2 | 0 | 0 | 5 |
| Rad-risk | 0 | 1 | 0 | 0 | 1 | 0 | 0 | 2 |
| GLCM: grey level co-occurrence matrix; GLRLM: grey-level run-length matrix; GLZLM: grey-level zone length matrix; ICC: intraclass correlation coefficient; NGLDM: neighborhood grey-level difference matrix; rad-risk: radiomic risk. ^a^ ICC_lb95%_, the lower bounds of the 95% confidence interval of the intraclass correlation coefficient value. | | | | | | | | |

**Supplementary Table 2. Reproducibility of the texture features.**

| **Imaging Indices** | **ICC** | **ICC_95%lb_** | **Category^a^** |
| --- | --- | --- | --- |
| SUVbwmin | 0.859 | 0.745 | Moderate |
| SUVbwmean | 0.976 | 0.954 | Excellent |
| SUVbwstd | 0.970 | 0.936 | Excellent |
| SUVbwmax | 0.975 | 0.956 | Excellent |
| SUVbwQ1 | 0.961 | 0.931 | Excellent |
| SUVbwQ2 | 0.973 | 0.946 | Excellent |
| SUVbwQ3 | 0.972 | 0.945 | Excellent |
| SUVbwSkewness | 0.869 | 0.787 | Good |
| SUVbwKurtosis | 0.859 | 0.771 | Good |
| SUVbwpeakSphere0.5mL | 0.986 | 0.977 | Excellent |
| SUVbwpeakSphere1mL | 0.992 | 0.987 | Excellent |
| CONVENTIONAL_TLG(mL) | 0.968 | 0.946 | Excellent |
| DISCRETIZED_SUVbwmin | 0.819 | 0.709 | Moderate |
| DISCRETIZED_SUVbwQ1 | 0.938 | 0.896 | Good |
| DISCRETIZED_SUVbwQ2 | 0.966 | 0.940 | Excellent |
| DISCRETIZED_SUVbwQ3 | 0.966 | 0.936 | Excellent |
| DISCRETIZED_SUVbwSkewness | 0.873 | 0.794 | Good |
| DISCRETIZED_SUVbwKurtosis | 0.870 | 0.788 | Good |
| SUVbwpeakSphere0.5mL | 0.986 | 0.977 | Excellent |
| Histogram_Skewness | 0.970 | 0.950 | Excellent |
| Histogram_Kurtosis | 0.964 | 0.939 | Excellent |
| Histogram_Entropy_log2 | 0.984 | 0.969 | Excellent |
| Histogram_Energy[=Uniformity] | 0.982 | 0.969 | Excellent |
| SHAPE_Volume(mL) = MTV | 0.956 | 0.920 | Excellent |
| SHAPE_Volume(vx) | 0.966 | 0.934 | Excellent |
| SHAPE_SPHERICITY | 0.707 | 0.539 | Moderate |
| SHAPE_Surface(mm2)[onlyFor3DROI] | 0.945 | 0.907 | Excellent |
| SHAPE_Compacity[onlyFor3DROI] | 0.944 | 0.629 | Moderate |
| GLCM_Homogeneity[=InverseDifference] | 0.985 | 0.940 | Excellent |
| GLCM_Energy[=AngularSecondMoment] | 0.984 | 0.973 | Excellent |
| GLCM_Contrast[=Variance] | 0.978 | 0.948 | Excellent |
| GLCM_Correlation | 0.879 | 0.409 | Poor |
| GLCM_Entropy_log2 | 0.987 | 0.978 | Excellent |
| GLCM_Dissimilarity | 0.981 | 0.938 | Excellent |
| GLRLM_SRE | 0.986 | 0.974 | Excellent |
| GLRLM_LRE | 0.972 | 0.953 | Excellent |
| GLRLM_LGRE | 0.995 | 0.992 | Excellent |
| GLRLM_HGRE | 0.968 | 0.930 | Excellent |
| GLRLM_SRLGE | 0.995 | 0.991 | Excellent |
| GLRLM_SRHGE | 0.968 | 0.932 | Excellent |
| GLRLM_LRLGE | 0.987 | 0.979 | Excellent |
| GLRLM_LRHGE | 0.966 | 0.918 | Excellent |
| GLRLM_GLNU | 0.951 | 0.879 | Good |
| GLRLM_RLNU | 0.961 | 0.924 | Excellent |
| GLRLM_RP | 0.983 | 0.970 | Excellent |
| NGLDM_Coarseness | 0.712 | 0.523 | Moderate |
| NGLDM_Contrast | 0.945 | 0.908 | Excellent |
| NGLDM_Busyness | 0.967 | 0.885 | Good |
| GLZLM_SZE | 0.677 | 0.505 | Moderate |
| GLZLM_LZE | 0.957 | 0.915 | Excellent |
| GLZLM_LGZE | 0.865 | 0.780 | Good |
| GLZLM_HGZE | 0.965 | 0.794 | Good |
| GLZLM_SZLGE | 0.497 | 0.274 | Poor |
| GLZLM_SZHGE | 0.964 | 0.804 | Good |
| GLZLM_LZLGE | 0.900 | 0.836 | Good |
| GLZLM_LZHGE | 0.984 | 0.973 | Excellent |
| GLZLM_GLNU | 0.935 | 0.872 | Good |
| GLZLM_ZLNU | 0.942 | 0.904 | Excellent |
| GLZLM_ZP | 0.963 | 0.937 | Excellent |
| ICC_95%lb_, the lower bounds of the 95% confidence interval of the intraclass correlation coefficient value; ^a^ Reproducibility of each feature was categorized as: poor, ICC_lb95%_ <0.50; moderate, ICC_lb95%_ of 0.50-0.75; good, ICC_lb95%_ of 0.75-0.90; excellent, ICC_lb95%_ ≥0.90. | | | |

**Supplementary Table 3. PET features for prediction of MYCN amplification.**

| **PET features** | **MYCN** | | ***p*-value** | **Adjusted *p-*value** | **AUCs** | ***p*-value** |
| --- | --- | --- | --- | --- | --- | --- |
|  | **Non-amplification** | **Amplification** |  |  |  |  |
| Conventional indices (n = 17) | | | | | | |
| SUVmean | 1.8 (0.9-6.2) | 2.7 (1.8-4.0) | <0.001 | 0.002 | 0.798 | <0.001 |
| SUVstd | 0.7 (0.2-4.9) | 1.2 (0.6-2.5) | <0.001 | <0.001 | 0.817 | <0.001 |
| SUVmax | 5.1 (1.6-26.5) | 7.9 (4.1-18.5) | <0.001 | 0.005 | 0.771 | <0.001 |
| SUVQ1 | 1.3 (0.1-2.7) | 1.7 (1.0-2.9) | 0.005 | 0.076 | 0.706 | 0.005 |
| SUVQ2 | 1.8 (0.6-5.5) | 2.5 (1.4-4.2) | <0.001 | 0.003 | 0.784 | <0.001 |
| SUVQ3 | 2.2 (1.0-9.4) | 3.7 (2.0-5.0) | <0.001 | <0.001 | 0.819 | <0.001 |
| SUV_Skewness | 0.6 (-0.5-2.7) | 0.4 (-0.4-3.2) | 0.120 | 0.436 | 0.614 | 0.120 |
| SUVKurtosis | 3.5 (2.3-11.7) | 2.6 (2.0-22.1) | 0.007 | 0.081 | 0.698 | 0.007 |
| SUVpeak_1mL | 3.9 (1.2-19.7) | 6.2 (3.5-10.4) | <0.001 | 0.003 | 0.792 | <0.001 |
| TLG | 283 (7-3959) | 933 (39-3793) | <0.001 | 0.002 | 0.776 | <0.001 |
| DISCRETIZED_ SUVQ1 | 5 (1-9) | 6 (4-10) | 0.009 | 0.081 | 0.689 | 0.010 |
| DISCRETIZED_ SUVQ2 | 6 (2-18) | 8.5 (5-14) | <0.001 | 0.005 | 0.772 | <0.001 |
| DISCRETIZED_ SUVQ3 | 8(2-30) | 12.5 (7-16) | <0.001 | 0.001 | 0.808 | <0.001 |
| DISCRETIZED_SUVSkewness | 0.6 (-0.5-2.7) | 0.4 (-0.4-3.1) | 0.145 | 0.436 | 0.607 | 0.145 |
| DISCRETIZED_SUVKurtosis | 3.5 (2.3-11.6) | 2.6 (2.1-21.5) | 0.009 | 0.081 | 0.692 | 0.009 |
| DISCRETIZED_SUVpeak | 14.0 (4.8-63.3) | 22.1 (12.4-37.6) | <0.001 | 0.004 | 0.781 | <0.001 |
| Histogram indices (n =4) | | | | | | |
| DISCRETIZED_Histogram_Skewness | 3.2 (1.4-6.7) | 1.9 (1.2-3.8) | <0.001 | <0.001 | 0.846 | <0.001 |
| DISCRETIZED_Histogram_Kurtosis | 12.4 (3.5-49.7) | 5.3 (2.7-16.6) | <0.001 | <0.001 | 0.853 | <0.001 |
| DISCRETIZED_Histogram_Entropy_log2 | 3.1 (0.8-5.6) | 3.9 (2.8-4.8) | <0.001 | <0.001 | 0.834 | <0.001 |
| DISCRETIZED_Histogram_Energy[=Uniformity] | 0.1 (0.0-0.4) | 0.1 (0.0-0.2) | <0.001 | <0.001 | 0.839 | <0.001 |
| Shape indices (n =3) | | | | | | |
| SHAPE_Volume(mL) | 140 (4-1127) | 472 (16-960) | 0.004 | 0.059 | 0.714 | 0.004 |
| SHAPE_Volume(vx) | 2982 (138-22654) | 9559 (330-31559) | 0.002 | 0.030 | 0.733 | 0.002 |
| SHAPE_Surface(mm2) | 18464 (1516-90558) | 42123 (3813-77992) | 0.006 | 0.077 | 0.704 | 0.006 |
| Second order texture indices (n = 27) | | | | | | |
| GLCM_Homogeneity[=InverseDifference] | 0.6 (0.3-0.7) | 0.5 (0.4-0.6) | <0.001 | 0.003 | 0.789 | <0.001 |
| GLCM_Energy[=AngularSecondMoment] | 0.0 (0.0-0.2) | 0.0 (0.0-0.0) | <0.001 | <0.001 | 0.814 | <0.001 |
| GLCM_Contrast[=Variance] | 4.1 (0.7-87.9) | 8.9 (4.2-28.1) | <0.001 | 0.002 | 0.797 | <0.001 |
| GLCM_Entropy_log2[=JointEntropy] | 5.5 (2.8-10.1) | 7.2 (5.3-8.4) | <0.001 | <0.001 | 0.822 | <0.001 |
| GLCM_Dissimilarity | 1.4 (0.6-6.3) | 2.2 (1.4-3.5) | <0.001 | 0.002 | 0.799 | <0.001 |
| GLRLM_SRE | 0.8 (0.7-0.9) | 0.9 (0.8-0.9) | 0.001 | 0.016 | 0.748 | 0.001 |
| GLRLM_LRE | 2.2 (1.3-10.1) | 1.7 (1.3-2.6) | 0.006 | 0.081 | 0.701 | 0.006 |
| GLRLM_LGRE | 0.0 (0.0-0.2) | 0.0 (0.0-0.1) | 0.009 | 0.081 | 0.692 | 0.009 |
| GLRLM_HGRE | 46.6 (11.7-694.2) | 105.6 (45.8-230.5) | <0.001 | <0.001 | 0.825 | <0.001 |
| GLRLM_SRLGE | 0.0 (0.0-0.1) | 0.0 (0.0-0.0) | 0.003 | 0.046 | 0.721 | 0.003 |
| GLRLM_SRHGE | 40.6 (8.6-664.4) | 95.8 (39.1-218.3) | <0.001 | <0.001 | 0.820 | <0.001 |
| GLRLM_LRLGE | 0.1 (0.0-8.9) | 0.0 (0.0-0.7) | 0.073 | 0.435 | 0.632 | 0.073 |
| GLRLM_LRHGE | 91.2 (33.4-840.5) | 170.0 (88.1-315.8) | <0.001 | 0.002 | 0.791 | <0.001 |
| GLRLM_GLNU | 282.6 (23.4-2025.9) | 506.6 (29.2-1715.4) | 0.043 | 0.340 | 0.649 | 0.043 |
| GLRLM_RLNU | 1507.1 (92.4-11994.9) | 5400.5 (252.9-20160.4) | <0.001 | 0.003 | 0.786 | <0.001 |
| GLRLM_RP | 0.8 (0.6-0.9) | 0.8 (0.7-0.9) | 0.002 | 0.035 | 0.728 | 0.002 |
| NGLDM_Contrast | 0.0 (0.0-0.5) | 0.1 (0.0-0.2) | <0.001 | 0.011 | 0.756 | <0.001 |
| NGLDM_Busyness | 2.9 (0.6-20.3) | 4.0 (0.6-10.8) | 0.741 | 0.900 | 0.524 | 0.741 |
| GLZLM_LZE | 5351.1 (46.7-101305.1) | 2547.8 (47.1-15467.4) | 0.091 | 0.436 | 0.624 | 0.091 |
| GLZLM_LGZE | 0.0 (0.0-0.2) | 0.0 (0.0-0.0) | <0.001 | 0.001 | 0.806 | <0.001 |
| GLZLM_HGZE | 63.9 (9.4-769.2) | 143.0 (57.9-330.0) | <0.001 | 0.002 | 0.800 | <0.001 |
| GLZLM_SZHGE | 32.4 (2.3-532.4) | 78.5 (25.9-217.3) | <0.001 | 0.002 | 0.796 | <0.001 |
| GLZLM_LZLGE | 199.3 (1.2-25719.6) | 84.2 (0.7-1315.9) | 0.070 | 0.435 | 0.634 | 0.070 |
| GLZLM_LZHGE | 116291 (3161-1869823) | 141222 (4051-2192841) | 0.900 | 0.900 | 0.491 | 0.900 |
| GLZLM_GLNU | 20.3 (2.6-124.0) | 75.2 (8.2-205.9) | <0.001 | 0.001 | 0.805 | <0.001 |
| GLZLM_ZLNU | 44.1 (1.2-2468.1) | 322.7 (19.6-1070.9) | <0.001 | <0.001 | 0.836 | <0.001 |
| GLZLM_ZP | 0.1 (0.0-0.43) | 0.12 (0.1-0.3) | 0.001 | 0.019 | 0.744 | 0.001 |
